# Application of point-of-care ultrasound for different types of esophageal foreign bodies: three case reports: A CARE-compliant article

**DOI:** 10.1097/MD.0000000000018893

**Published:** 2020-01-24

**Authors:** Jung Hwan Ahn, Youdong Sohn

**Affiliations:** a Department of Emergency Medicine, Ajou University School of Medicine, Suwon; b Department of Emergency Medicine, Seoul Metropolitan Government – Seoul National University Boramae Medical Center, College of Medicine, Seoul National University, Seoul, Republic of Korea; c Department of Emergency Medicine, Sheikh Khalifa Specialty Hospital, Ras Al Khaimah, UAE.

**Keywords:** esophagus, food, foreign body, pill, ultrasound

## Abstract

**Rationale:**

Esophageal point-of-care ultrasound (POCUS) has recently been reported as a useful, quick, safe, and simple technique to detect esophageal foreign bodies (FBs). However, case series to detect esophageal FB using POCUS have been rarely reported. Chicken bones and pills, especially, have not yet been reported as esophageal FBs. The objective of this case series was to describe the POCUS findings of 3 different materials—food, pill, and chicken bone.

**Patient concerns:**

Case 1, a 75-year-old woman with odynophagia and neck pain occurring 30 min after eating chicken porridge; Case 2, a 32-year-old woman with neck discomfort occurring 2 h after taking a pill; Case 3, a 29-year-old woman reporting FB sensation in the neck that occurred 1 h after eating sausage and rice soup.

**Diagnosis:**

Case 1. Cervical esophageal FB (chicken bone), Case 2. Cervical esophageal FB (oral pill), Case 3. Cervical esophageal FB (food).

**Interventions:**

Case 1. POCUS, urgent esophagogastroduodenoscopy (EGD) with alligator forceps. POCUS findings; hyperechoic material (suspected FB) that did not disappear by swallowing and esophageal dilatation with pooling of secretions. Case 2. POCUS. POCUS findings; hypoechoic material (suspected FB) that did not disappear by swallowing, and esophageal bulging above the FB, especially observed in the longitudinal view. Case 3. POCUS. POCUS findings; hyperechoic material (suspected FB) with reverberation artifact that did not disappear with swallowing efforts. Prior FB esophageal bulging with persistent air-fluid level was especially observed in the longitudinal view.

**Outcomes:**

Case 1. FB was removed by EGD with alligator forceps. Case 2. Symptoms disappeared under observation without EGD. Follow-up POCUS revealed normalized bulging esophagus. Case 3. These symptoms improved after vomiting a large piece of food material. Three patients were discharged without complications.

**Lessons:**

In this case series, the impacted materials were chicken bone, pill, and food. However, POCUS findings were similar (esophageal dilation, hyperechoic or hypoechoic lesion with mixed echogenic contents in food or secretion, and no change with swallowing efforts). A longitudinal view was useful to assume the presence of cervical esophageal FB in all three cases. Thus, POCUS findings could be indirect signs of a FB in the esophagus.

## Abstract 2

Supplemental Digital Content is available in the text

## 1 Introduction

Point-of-care ultrasound (POCUS) is considered as one of the major areas for accessing more accurate diagnosis by narrowing the differentials, providing the guidance of treatment, and/or guidance of procedure.^[1,2]^ POCUS has been widely used to detect radiolucent or radiopaque foreign body (FB) in skin and soft tissue.^[3,4]^ Recently, esophageal POCUS has been reported to be a useful, quick, safe, and simple technique to detect esophageal FB.^[2,4–9]^ However, the detection of esophageal FB by using POCUS has been rarely reported. To the best of our knowledge, detection of esophageal FB by using POCUS has only been reported in 9 cases of children and 5 cases of adults so far.^[2,4–9]^ Especially, all 5 cases of adults were about radiolucent food material impaction.^[2]^ Cases of detecting esophageal FB such as oral pill and chicken bone by using POCUS have not been reported based on Medline literature search.

Herein, we report three cases of detection of esophageal FB (chicken bone, pill, and food impaction) by using POCUS in adults. The present case series describe POCUS findings of three different materials (food impaction, pill, and chicken bone).

Patients have provided informed consent for publication of the current case report. The approval (IRB no. 10-2019-38) of the Institutional Review Board of Seoul Metropolitan Government—Seoul National University Boramae Medical Center, Seoul, Republic of Korea was obtained.

## 2 Case report

### 2.1 Case 1

**Figure 1:**
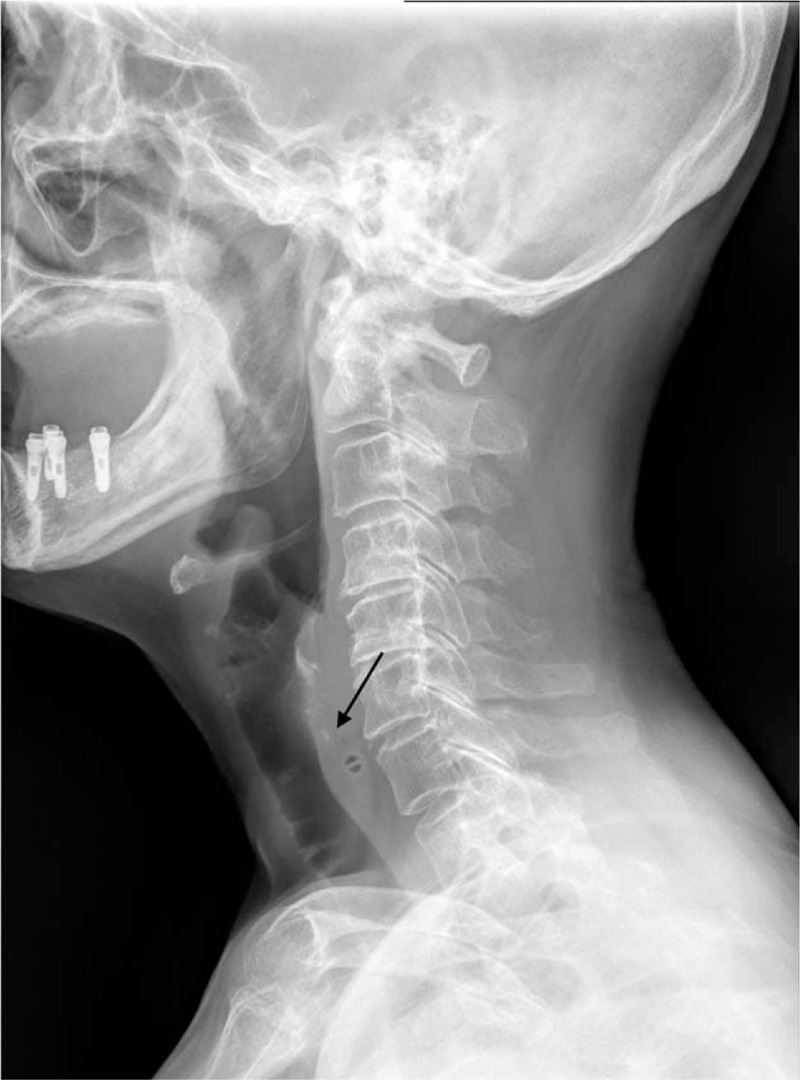
A 75-year-old woman (case 1) with complaint of odynophagia and neck pain that occurred 30 min after eating chicken porridge. Simple neck lateral radiography showing radiopaque material suspected as FB (black arrow). FB = foreign body.

**Figure 2:**
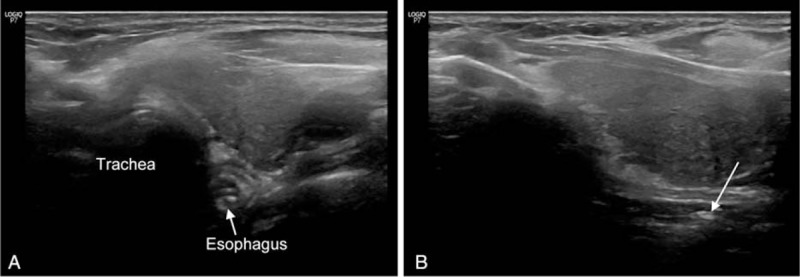
A 75-year-old woman (case 1) with complaint of odynophagia and neck pain that occurred 30 min after eating chicken porridge. POCUS view of chicken bone. (A) Axial view showing dilated esophagus and hyperechoic material suspected as FB (white arrow). (B) Longitudinal view showing hyperechoic material suspected as FB (white arrow). FB = foreign body, POCUS = point-of-care ultrasound.

A 75-year-old woman with a past history of hypertension visited emergency department (ED) with complaint of odynophagia and neck pain that occurred 30 min after eating chicken porridge. There was no fever or dyspnea upon arrival to ED. Review of system, physical examination, and laboratory findings were unremarkable except odynophagia and pain in the upper anterior neck. On simple radiography (neck lateral view), esophageal FB was suspected but not definitively identified (Fig. 1). Therefore, the emergency physician and gastroenterologist planned to perform a neck computed tomography (CT) to confirm the location of FB and rule out the possibility of perforation. Before performing neck CT, POCUS was performed to detect the esophageal FB by an attending emergency physician with experience of POCUS for 10 years and a faculty in World Interactive Network Focused on Critical Ultrasound (WINFOCUS) group. POCUS revealed a hyperechoic material suspected of FB and esophageal dilatation with pooling of secretions. It did not disappear by swallowing efforts (Fig. 2, see Video, Supplemental Video 1, which demonstrates POCUS findings of chicken bone. (A) Transverse view. (B) Longitudinal view). Urgent esophagogastroduodenoscopy (EGD) was performed. During EGD, chicken bone was observed at 20 cm from the upper incisor. During attempt to remove the FB with alligator forceps, the patient vomited and the chicken bone was self-removed. Two hours after the EGD, the patient was discharged from ED without any complication.

### 2.2 Case 2

**Figure 3:**
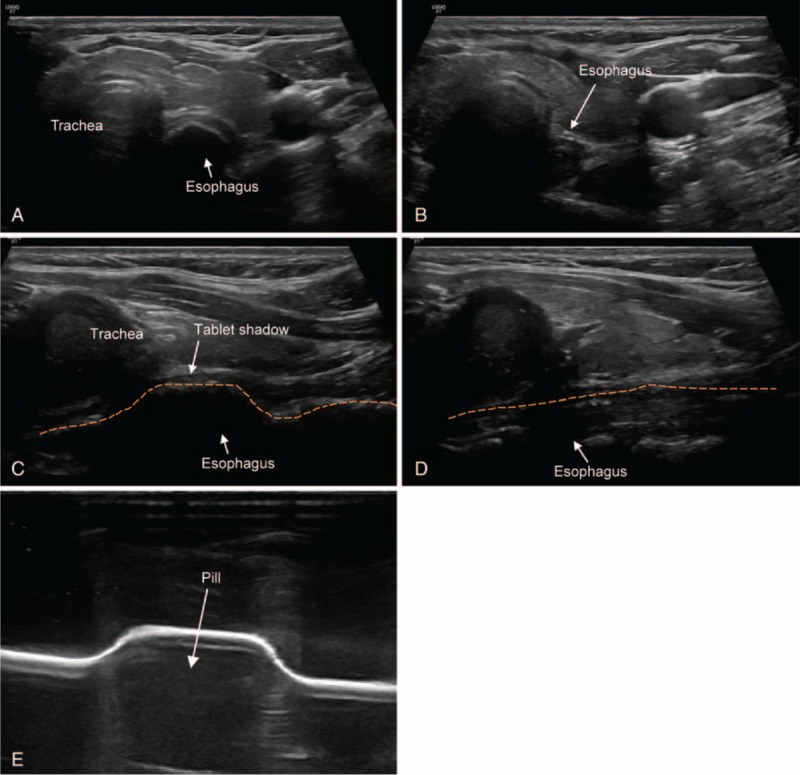
A 32-year-old woman (case 2) with discomfort on neck that occurred 2 h after taking a pill. The white arrow in each figure indicates the structure such as trachea and esophagus. The name of the structures is written at the end of the arrow or above the structure. (A) Axial view showing bulging esophagus due to impaction of pill. (B) Axial view showing collapsed esophagus after the pill went down. (C) Longitudinal view showing focal bulging esophagus due to impaction of pill. Dash line outlines the esophagus. (D) Longitudinal view showing collapsed esophagus after the pill went down. Dash line outlines the esophagus. (E) Simulated sonographic image of a pill. After the latex glove was filled with water, a pill was placed under the glove and the probe was placed on the glove to obtain a sonographic image of the pill. The pill was shown as hypoechoic lesion in the ultrasound.

A 32-year-old woman was admitted to ED with discomfort on the neck that occurred 2 h after taking a pill orally. On arrival to ED, she heard sounds of air bubbles coming from her neck when she drank water. She had no dyspnea or fever. Review of system and physical examination were unremarkable except discomfort on her neck. Simple radiography (neck lateral view) did not provide specific findings. Therefore, the emergency physician planned to observe the passage of pills. Otherwise, EGD was planned. During observation, POCUS was performed to detect the esophageal FB by an attending emergency physician with experience of POCUS for 10 years and a faculty in WINFOCUS group. POCUS revealed hypoechoic material suspected FB which did not disappear by swallowing with esophageal bulging above FB which was especially observed in longitudinal view (Fig. 3). While observing, her symptoms disappeared. Follow-up POCUS revealed that the bulging esophagus was normalized (Fig. 3). The patient was discharged from ED without any complication.

### 2.3 Case 3

**Figure 4:**
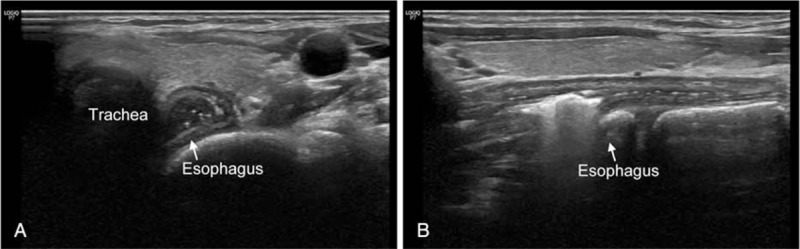
A 29-year-old woman (case 3) with FB sensation on neck that occurred 1 h after eating sausage and rice soup. The white arrow in each figure indicates the structure such as trachea and esophagus. The name of the structures is written at the end of the arrow or above the structure. (A) Axial view showing bulging esophagus with pooling of secretion and hyperechoic material suspected as food. (B) Longitudinal view showing hyperechoic material suspected as FB with reverberation artifact that did not disappear by swallowing efforts and esophageal bulging. FB = foreign body.

A 29-year-old woman visited ED with complaint of FB sensation on her neck that occurred 1 h after eating sausage and rice soup. Review of system and physical examination were unremarkable except discomfort on her neck. Simple radiography (neck lateral view) did not provide specific findings. POCUS was performed to detect the esophageal FB by an attending emergency physician with experience of POCUS for 10 years and a faculty in WINFOCUS group. POCUS revealed hyperechoic material suspected of FB with reverberation artifact which did not disappear by swallowing efforts. Prior FB esophageal bulging with persistent air-fluid level was especially observed in longitudinal view (Fig. 4, see Video, Supplemental Video 2, which demonstrates POCUS findings of food material. Longitudinal view). These symptoms improved after vomiting of a large food material. Eventually, the patient was discharged from ED without any complication.

## 3 Discussion

Imaging such as simple radiography and/or CT plays a key role in detecting and diagnosing esophageal FB.^[10]^ The majority of FB in adults are food boluses (34–59%) or bones (16–18%) followed by dental prostheses, pills, coins, and batteries.^[2,6,11]^ The sensitivity of simple radiography has been reported to be ranging from 42% to 80%.^[12,13]^ CT is widely used to detect esophageal FB. It has a sensitivity of 94.7% to 100% regardless of characteristics of FB.^[13,14]^ However, it is well known that CT is the last choice to diagnose diseases if there is another diagnostic modality that can replace CT due to radiation induced malignancy.^[15]^ Nowadays, POCUS has been introduced as a simple and non-invasive diagnostic modality without problem of radiation exposure.^[2,4–9]^ In addition, it is a useful tool to detect radiolucent objects.^[2,4–9]^

As mentioned above, although POCUS has been introduced as a useful tool for the diagnosis of cervical esophageal FB, case series for detecting cervical esophageal FB by using POCUS has been only reported in 9 children and 5 adults.^[2,4–9]^ In previous case series of food impaction in 5 adults, POCUS findings of cervical esophageal FB have been reported as esophageal dilation, hyperechoic lesion with various associated artifacts such as reverberation, mixed echogenic contents in food, and no change with swallowing efforts.^[2]^ In the previous case series report, all subjects (5 patients) had POCUS finding of dilated esophagus with persistent air–fluid level that did not disappear by swallowing efforts.^[2]^ The finding of the direct visualized esophageal FB was observed in 2 out of 5 patients.^[2]^ These POCUS findings of impacted food material described in the previous case series were also observed in food impaction of our case series. Interestingly, in case 3 about food impaction of our case series, all POCUS findings were better observed in longitudinal view than in axial view as seen in the video clip (see Video, Supplemental Video 2, longitudinal view).

POCUS images of chicken bone (case 1) and pill (case 2) in esophagus had an interesting point that have not been reported in previous case reports. Unlikely our expectation that chicken bone was well recognized in POCUS such as hyperechoic lesion with acoustic shadow like coin or metal ring, it was difficult to observe the full length of chicken bone and the shadow. Interestingly, esophageal dilation, mixed echogenic contents in secretion and/or food, and no change with swallowing efforts like POCUS findings of food impaction were more easily observed than ultrasound image of bone. The reasons might be as follows:

1. pooling of secretion caused by swelling of the esophagus as a result of irritation caused by chicken bone and swelling itself might make POCUS image look like food impaction; and
2. the shape and direction of the bone stuck in the esophagus might not coincide with the direction of the probe.

The previous case report of impacted bone marrow in esophagus in child has reported POCUS findings of dilated esophagus by mixed echogenicity contents and hypoechoic lesion suspected as FB.^[8]^ The pill in our case series was seen as hypoechoic lesion with dilated esophagus. Also, the longitudinal view was useful for detecting the cervical esophageal FB, the same as case 3 about food impaction. We conducted ultrasound simulation to obtain simulated sonographic image of a pill to confirm that the ultrasound finding of a pill looked hypoechoic. Simulation test was conducted using a latex glove. After the latex glove was filled with water, a pill was placed under the glove and the probe was placed on the glove to obtain a simulated sonographic image of a pill. Figure 3E shows the simulated sonographic image of a pill as hypoechoic lesion like our case.

In this case series, impacted materials were different (chicken bone, pill, and food). However, their POCUS findings were similar, showing esophageal dilation, hyperechoic or hypoechoic lesion with mixed echogenic contents in food or secretion, and no change with swallowing efforts. We believe that these POCUS findings would be powerful indirect signs of FB in esophagus. Based on this case series, the authors thought that a longitudinal view might be useful for determining the presence of cervical esophageal FB. In Case 1, involving a chicken bone, it was difficult to accurately image the shadow of the bone. However, indirect signs of esophageal FBs and hyperechoic lesions suspected as FBs were easily observed in the longitudinal view. In Case 2 involving a pill, esophageal dilation was clearly observed in the longitudinal view. Also, in Case 3 involving food impaction, the longitudinal view was useful for detecting indirect signs of FBs in the upper esophagus. To the best of our knowledge, there have been no case reports discussing the usefulness of the longitudinal view for detecting cervical esophageal FBs. One case report stated that the longitudinal view is useful for detecting FBs in the lower esophagus, especially at the gastroesophageal junction.^[2]^ Based on the observation of our case series, we carefully suggest that scanning of the longitudinal view in combination with the axial view might allow clinicians to more confidently determine the presence of cervical esophageal FBs than using only the axial view. Although POCUS is not a standard method to detect esophageal FB, these findings from the present case series provide an evidence that cervical esophageal POCUS is very useful tool for detecting esophageal FB.

## Author contributions

**Conceptualization:** Jung Hwan Ahn, Youdong Sohn.

**Data curation:** Jung Hwan Ahn, Youdong Sohn.

**Investigation:** Jung Hwan Ahn, Youdong Sohn.

**Methodology:** Jung Hwan Ahn, Youdong Sohn.

**Project administration:** Jung Hwan Ahn.

**Supervision:** Youdong Sohn.

**Writing – original draft:** Jung Hwan Ahn.

**Writing – review & editing:** Youdong Sohn.

Youdong Sohn: 0000-0001-8789-0090.

Youdong Sohn orcid: 0000-0001-8789-0090.

## Supplementary Material

Supplemental Digital Content

## Supplementary Material

Supplemental Digital Content
